# Selected polyphenols potentiate the apoptotic efficacy of glycolytic inhibitors in human acute myeloid leukemia cell lines. Regulation by protein kinase activities

**DOI:** 10.1186/s12935-016-0345-y

**Published:** 2016-09-07

**Authors:** Elena de Blas, María Cristina Estañ, María del Carmen Gómez de Frutos, Javier Ramos, María del Carmen Boyano-Adánez, Patricio Aller

**Affiliations:** 1 Centro de Investigaciones Biológicas (CIB), Consejo Superior de Investigaciones Científicas (CSIC), Ramiro de Maeztu 9, 28040 Madrid, Spain; 2 Departamento de Biología de Sistemas, Unidad de Bioquímica y Biología Molecular, Facultad de Medicina y Ciencias de la Salud, Universidad de Alcalá, Alcalá de Henares, Madrid, Spain; 3 Escuela Técnica Superior de Ingenieros Agrónomos, Universidad Politécnica, Madrid, Spain; 4 Instituto de Investigaciones Biomédicas Alberto Sols, Consejo Superior de Investigaciones Científicas, Universidad Autónoma de Madrid, Madrid, Spain

**Keywords:** 2-Deoxy-d-glucose, Lonidamine, Natural polyphenols, Apoptosis, Oxidative stress, Protein kinases (Akt, ERKs), Acute myeloid leukemia cells

## Abstract

**Background:**

The glycolysis inhibitor 2-deoxy-d-glucose (2-DG) is a safe, potentially useful anti-tumour drug, but its efficacy is normally low when used alone. Recent studies indicated that 2-DG stimulates the PI3K/Akt and MEK/ERK defensive pathways, which limits the apoptotic efficacy in tumour cell lines. We hypothesized that co-treatment with selected polyphenols could improve 2-DG-provoked apoptosis by preventing defensive kinase activation.

**Methods:**

Cell proliferation was measured by cell counting or the MTT assay. Cell cycle, apoptosis and necrosis were determined by propidium iodide staining and/or annexin V labeling followed by flow cytometry. Mitochondria pore transition and depolarization were determined by calcein-ATM or rhodamine 123 labeling followed flow cytometry. Intracellular reactive oxygen species and GSH were determined by dichlorodihydrofluorescein diacetate or monochlorobimane labeling followed by flow cytometry or fluorimetry. Expression and phosphorylation of protein kinases were analyzed by the Western blot.

**Results:**

(i) 2-DG-provoked apoptosis was greatly potentiated by co-treatment with the sub-lethal concentrations of the flavonoid quercetin in human HL60 acute myeloblastic leukemia cells. Allowing for quantitative differences, apoptosis potentiation was also obtained using NB4 promyelocytic and THP-1 promonocytic cells, using curcumin or genistein instead of quercetin, and using lonidamine instead of 2-DG, but not when 2-DG was substituted by incubation in glucose-free medium. (ii) Quercetin and 2-DG rapidly elicited the opening of mitochondria pore transition, which preceded the trigger of apoptosis. (iii) Treatments did not affect GSH levels, and caused disparate effects on reactive oxygen species generation, which did not match the changes in lethality. (iv) 2-DG and lonidamine stimulated defensive Akt and ERK phosphorylation/activation, while glucose starvation was ineffective. Polyphenols prevented the stimulation of Akt phosphorylation, and in some cases also ERK phosphorylation. In addition, quercetin and 2-DG stimulated GSK-3α,β phosphorylation/inactivation, although with different isoform specificity. The use of pharmacologic inhibitors confirmed the importance of these kinase modifications for apoptosis.

**Conclusions:**

The present in vitro observations suggest that co-treatment with low concentrations of selected polyphenols might represent a manner of improving the poor anti-tumour efficacy of some glycolytic inhibitors, and that apoptosis potentiation may be at least in part explained by the regulation of defensive protein kinase activities.

**Electronic supplementary material:**

The online version of this article (doi:10.1186/s12935-016-0345-y) contains supplementary material, which is available to authorized users.

## Background

A characteristic of tumour cells is the profound re-organization of metabolic parameters in relation to their healthy counterparts, allowing them to obtain the macromolecular constituents required for their rapid de-regulated growth and also as alternative sources of energy [1–3]. One of the best known modifications is the increased dependence on glucose metabolism instead of oxidative phosphorylation, even under aerobic conditions (a property known as “aerobic glycolysis” or “Warburg’’ effect). This peculiarity made possible the development of glycolysis-targeting drugs as potential anti-cancer agents. This category includes, among others, the glucose inactive analog 2-deoxy-d-glucose (2-DG) [4], the indazole derivative lonidamine (Lon) [5], and the small alkylating drug 3-bromopyruvate (3-BrP) [6]. Allowing for the disparity in chemical structure and hence in biochemical and molecular effects, these drugs target critical enzymes in the glycolytic pathway, namely hexokinase II (HKII) in the case of 2-DG and Lon [5, 7], and glyceraldehyde-3-phosphate dehydrogenase (GAPDH) and to a lower extent HKII in the case of 3-BrP [8]. While promissory results have been obtained in clinical assays [5, 6, 9, 10], the efficacy of these agents is different. Thus, 3-BrP is quite toxic per se, while 2-DG and Lon are well tolerated but poorly efficacious in monotherapy. Nonetheless, 2-DG and Lon may be useful as radio- and chemo-sensitizing agents, overcoming resistance and increasing cyto-reduction by conventional anti-tumour treatments [4, 5, 9]. Using combinatory assays with the anti-leukemic agent arsenic trioxide (Trisenox), we recently demonstrated that a common effect of the anti-glycolytic drugs is the stimulation (albeit with different kinetics and intensity) of Akt/mTOR and MEK/ERK defensive pathways in several human acute myeloid leukemia (AML) cell lines, and that this stimulation restrains the apoptotic efficacy of 2-DG and Lon when used as single agents [11, 12]. Akt and/or ERK activation by 2-DG was also observed in other tumour cell models [13–15].

Polyphenols represent a large collection of molecules present in the plant kingdom. At the low doses attainable in the daily diet these compounds exert multiple protective functions (e.g., against cellular oxidation, inflammation, aging, tumour initiation…). On the other hand, at high albeit still pharmacologically attainable concentrations many polyphenols selectively induce apoptosis in tumour cells, and exhibit clinical efficacy either alone or in combination with conventional anti-cancer drugs [16]. While the multiplicity of biochemical actions makes impossible to unequivocally ascribe their anti-cancer action to a single mechanism, a frequent effect of polyphenols is the inhibition of the PI3K/Akt defensive pathway [17, 18]. For instance, the flavonoid quercetin (Quer) is the natural analog of 2-(4-Morpholinyl)-8-phenyl-4H-1-benzopyran-4-one (LY294002), a potent PI3K inhibitor commonly used in laboratory research [19]. We previously observed that prolonged treatment (24–48 h) with sub-lethal concentrations of Quer, curcumin (Cur) and genistein (Gen) reduced the constitutive Akt phosphorylation in U937 and HL60 AML cells [20–22]. On this ground, we hypothesized the pre-treatment with polyphenols might prevent Akt activation and as a consequence improve the lethality of glycolytic inhibitors. With this hypothesis in mind, in the present work we analyze the capacity of Quer, Cur and Gen to cooperate with 2-DG and Lon to induce apoptosis in HL60 and other AML cell lines. The regulatory function of Akt and other protein kinases, as well as the potential importance of other factors such as mitochondrial dysfunction and oxidative stress, are examined.

## Methods

### Reagents and antibodies

All components for cell culture were obtained from Invitrogen, Inc. (Carlsbad, CA). Dichlorodihydrofluorescein diacetate (H_2_DCFDA) and monochlorobimane were obtained from Molecular Probes, Inc. (Eugene, OR). The kinase inhibitors 1,4-Diamino-2,3-dicyano-1,4-*bis*(2-aminophenylthio)butadiene (U0126), 2′-Amino-3′-methoxyflavone (PD98059), 2-(4-Morpholinyl)-8-phenyl-4H-1-benzopyran-4-one (LY294002), 5-Dihydro-5-methyl-1-β-d-ribofuranosyl-1,4,5,6,8-pentaazaacenaphtylen-3-amine hydrate (triciribine hydrate, Akt inhibitor V), 4-(4-Fluorophenyl)-2-(4-methylsulfinylphenyl)-5-(4-pyridyl)-1H-imidazole (SB203580), and the caspase inhibitor Z-Val-Ala-Asp(OMe)-CH_2_F (z-VAD-fmk), were obtained from Calbiochem (Darmstad, Germany), and 1-5-*tert*-Butyl-2-*p*-tolyl-2*H*-pyrazol-3-yl)-3-[4-(2-morpholin-4-yl-ethoxy)naphthalene-1-yl] urea (BIRB 796) from Shelleck (Houston, TX). Rabbit anti-human p44/42 MAP kinase, phospho-p44/p42 MAP kinase (Thr202/Tyr204), Akt, phospho-Akt (Ser473) (D9E) XP™, p38 MAP kinase, phospho-p38 MAP kinase (Thr180/Tyr182), phospho-GSK-3α/β (Ser21/9), and phospho-S6 ribosomal protein (Ser235/236) polyclonal antibodies, were obtained from Cell Signaling Technology Inc. (Danvers, MA). Mouse GSK-3α/β monoclonal antibody (0011-A) was obtained from Santa Cruz Biotechnology, Inc. (Santa Cruz, CA). Peroxidase-conjugated immunoglobulin G antibodies were from DAKO Diagnostics, S.A. (Barcelona, Spain). All other non-mentioned reagents and antibodies were from Sigma (Madrid, Spain).

### Cells and treatments

HL60 myeloblastic cells [23] and THP-1 promononocytic cells [24] were obtained from our institutional repository (CIB), and NB4 promyelocytic cells [25] were kindly supplied by Profs. M.D. Delgado and J. León (Departamento de Biología Molecular, Facultad de Medicina, Universidad de Cantabria, Santander, Spain). These cell lines represent distinct subtypes of human AML cells (HL60, M2; NB4, M3; THP-1, M5, according to the classification of the French-American-British (FAB) cooperative group), with substantial differences in molecular and biochemical parameters, and hence in the capacity of response to anti-cancer agents. Absence of mycoplasma contamination, and authentication by STR analysis, specific antigen expression, and PML-RARα fusion protein expression (NB4 cells) were corroborated by us or our technical staff. Cell handling (and all experimental procedures in general) was carried out strictly following the regulations of the Bioethics and Biosafety Commission of our Institution (Centro de Investigaciones Biológicas, CSIC). Conditions of cell growth and treatment were described in detail in preceding publications [11, 12]. For glucose starvation (Glu−), cells were extensively washed with phosphate-buffered saline (PBS) and then seeded at the appropriate concentration in glucose-lacking RPMI medium supplemented with 10 % (v/v) serum. For good comparison, the corresponding controls were subject to the same manipulation, but finally seeded in complete medium (Glu+).

Calcein-AM was commercially obtained as a 4 mM solution in dimethyl sulfoxide. Rhodamine 123 (R123, 1 mg/ml) was prepared in ethanol. Stock solutions of Lon (100 mM), Quer (100 mM), Gen (50 mM), Cur (20 mM), H_2_DCFDA (5 mM), monochlorobimane (200 mM), U0126 (2.63 mM), PD98059, LY294002 and triciribine (20 mM each), SB203580 (13,2 mM), SB216763 (50 mM), BIRB 796 (0.1 mM) and z-VAD-fmk (25 mM) were prepared in dimethyl sulfoxide. 3(4,5-Dimethyl-2-thiazolyl)-2,5-diphenyl-2*H*-tetrazolium bromide (MTT) was dissolved at 5 mg/ml in PBS. All these solutions were stored at −20 °C. A stock solution of propidium iodide (PI, 1 mg/ml) was prepared in phosphate buffered saline (PBS), and stored at 4 °C. 2-DG and DL-buthionine-(*S,R*)-sulfoximine (BSO) were freshly prepared at 250 and 50 mM, respectively, in PBS. 3-Bromopyruvate was freshly prepared at 30 mM in PBS, and the pH of the solution was adjusted at 7.2 with NaOH.

### Flow cytometry

The analysis of samples was carried out on an EPICS XL flow cytometer (Coulter, Hialeah, FL) equipped with an air-cooled argon laser tuned to 488 nm. The specific fluorescence signal corresponding to fluorescein isothiocyanate, H_2_DCFDA, calcein-AM and R123 was collected with a 525-nm band pass filter, and the signal corresponding to PI with a 620-nm band pass filter. A total of 10^4^ cells were scored in each determination.

### Cell proliferation, cell cycle, apoptosis and necrosis

Total cell proliferation was measured by cell counting of trypan-blue excluding cells, or by means of the MTT colorimetric assay. This later procedure gives an indirect estimation of the relative number of viable cells in the culture, based on changes in mitochondrial metabolic activity. Cell cycle phase distribution was routinely determined by cell permeabilization followed by PI staining and flow cytometry analysis. When convenient, the resulting histograms were analyzed with the FlowLogic program (Inivai, Victoria, Canada). This technique also provided an estimation of the frequency of apoptotic cells, characterized by low (sub-G_1_) DNA content. The criterion used for necrosis was the loss of plasma membrane integrity, as determined by free PI uptake into non-permeabilized cells and flow cytometry analysis. In addition, apoptosis and necrosis were determined simultaneously by double labeling with annexin V-FITC and PI followed by flow cytometry measurement using an Annexin V-FITC Apoptosis Detection Kit (Immunostep, Salamanca, Spain). This procedure allows the distinction between viable cells (annexin V-negative/PI-negative), early apoptotic cells (annexin V-positive/PI-negative), late apoptotic or necrotic cells (annexin V-positive/PI-positive), and genuine necrotic cells (annexin-V negative/PI-positive). Since the loss of plasma membrane integrity leading to free PI penetration is compatible with both genuine necrosis and late apoptosis (also termed “secondary” necrosis), the pan-caspase inhibitor z-VAD-fmk was occasionally used to discriminate between these two possibilities. A detailed description of all these techniques can be found in our preceding works [21, 26, and references therein].

### Mitochondrial membrane permeabilization and membrane potential dissipation

Inner mitochondrial membrane permeabilization (mIMP) was determined using the calcein-AM/CoCl_2_ method, originally reported by Petronilli et al. [27]. Our adaptation for flow cytometry using HL60 cells was already described in a preceding article [11]. Mitochondrial membrane potential (ΔΨm) was determined using the cationic agent R123 and flow cytometry analysis, as previously described [26].

### Reactive oxygen species and reduced glutathione levels

The intracellular accumulation of reactive oxygen species (ROS) was measured by flow cytometry using the ROS-sensitive probe H_2_DCFDA. The intracellular level of reduced glutathione (GSH) was measured in a Varioskan Flash microplate reader (Thermo Fisher Scientific Inc, Waltham, MA) at excitation wavelength of 390 nm and emission wavelength of 520 nm, using the fluorescent probe monochlorobimane. The detailed procedures were described in a previous publication [26].

### Immunoblotting

Cells were collected by centrifugation, washed with PBS and total protein extracts were obtained by lysing them for 20 min at 4 °C in a buffer consisting of 20 mM Tris–HCl (pH 7.5) containing 137 mM NaCl, 2 mM EDTA, 10 % (v/v) glycerol, and 1 % Nonidet P-40, and supplemented with a protease inhibitor cocktail, 1 mM sodium orthovanadate, and 10 mM NaF. After brief sonication and centrifugation for 15 min at 10,000×*g* at 4 °C, the supernatants were collected, and samples containing equal amounts of proteins were resolved by SDS–polyacrylamide gel electrophoresis. The proteins were then transferred to polyvinylidene fluoride (PVDF) membranes and immunodetected, as previously described [28]. When convenient, the relative band intensities were quantified using the Quantity One 1-D Analysis Software, version 4.6 (Bio-Rad Laboratories, Inc., Hercules, CA).

### Data analysis and presentation

Except when indicated, all experiments were repeated at least three times, and as a rule the results are expressed as the mean value ± SD. Statistical analyses were carried out using one way ANOVA with Dunnett or Bonferroni post-test, using SAS version 9.4 (SAS Institute, Cary NC). The Dunnett’s method was followed when comparing different treatments with controls, and Bonferroni’s when pairwise comparisons were performed. The symbols used were: ^&^, to compare treatment vs. control; *, to compare pairs of single treatments; and ^#^, to indicate that the value in a combined treatment is higher than the sum of values in the corresponding single treatments. Sum of values were obtained by considering single treatment as independent random variables. In all cases, single symbol means p < 0.05, double symbol p < 0.01, and triple symbol p < 0.001. n.s., non-significant.

## Results

### Cell proliferation and cell death

**Fig. 1:**
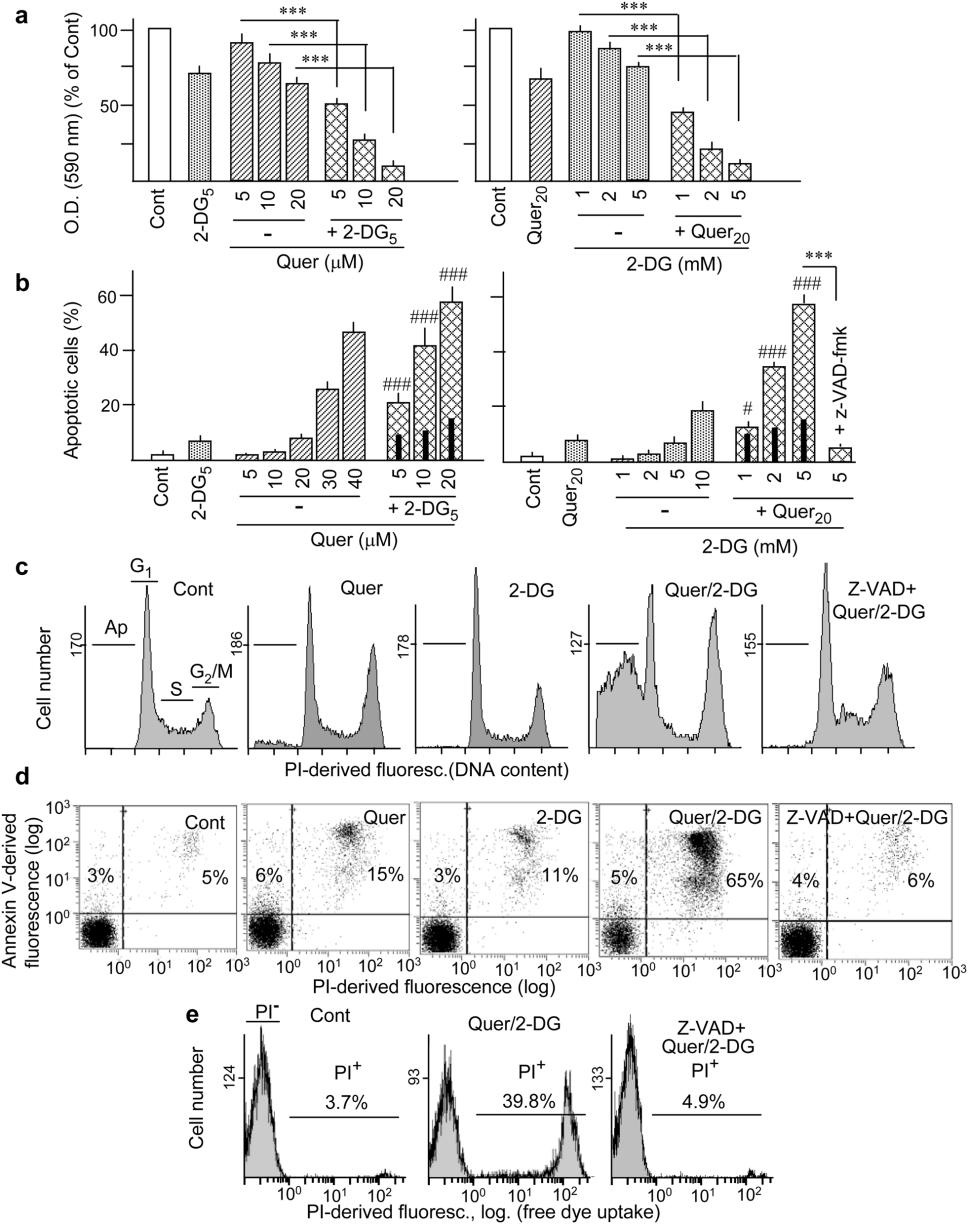
Effect of quercetin and 2-deoxy-d-glucose on cell viability and apoptosis generation. HL60 cells were either kept untreated (Cont) or treated with quercetin (Quer) and 2-deoxy-d-glucose (2-DG), alone and in combination. In the combined treatments the cells were pre-treated with Quer for 2 h, followed by addition of 2-DG for 24 h more, and with the occasional presence of the pan-caspase inhibitor z-VAD-fmk (50 μM). Drug concentrations are indicated as subheadings (Quer, μM; 2-DG, mM). When nothing is indicated, Quer was used at 20 μM and 2-DG at 5 mM. **a** Changes in cell viability, as evidenced by the MTT assay. Absorption values are expressed in relation to the control. **b** Frequency of apoptosis, represented by the sub-population of cells with sub-G_1_ DNA content obtained by flow cytometry. Examples of histograms showing cell cycle phases (G_1_, S and G_2_/M) and sub-G_1_ (Ap) are presented in **c**. **d** Frequency of early and late apoptotic cells, measured by cell surface annexin V binding and propidium iodide (PI) exclusion or uptake, respectively. **e** Flow cytometry histograms showing the frequency of PI-permeable cells, indicating plasma membrane disruption. The bar charts in (**a**–**b**) represent the mean ± S.D. of at least three determinations, measured by duplicate. The histograms in (**d**, **e**) are representative of one out of three determinations with similar results. Symbols mean: (*) significant differences between single treatments; (^#^) significant differences between the combined treatment and the sum of values in the corresponding individual treatments (e.g., co-incubation with 20 μM Quer and 5 mM 2-DG, in relation to the sum of 20 μM Quer alone plus 5 mM 2-DG alone) (*n.s.* non-significant). To better discern differences, in this case the sum of values in individual treatments is indicated by a thick black bar within the bar corresponding to the combined treatment. Single symbol, p < 0.05; double symbol, p < 0.01; triple symbol, p < 0.001

Firstly, we examined the capacity of Quer and 2-DG, alone and in combination, to affect proliferation activity and induce apoptosis at 24 h of treatment in HL60 cells. Because of the hypothesis advanced in the ‘‘Background’’ section, namely that polyphenols might prevent early regulatory gene responses elicited by metabolic inhibitors, in the combined treatments Quer was applied 2 h before 2-DG (and except when otherwise indicated, this procedure will be also followed in all experiments along the whole work). As shown in Fig. 1a, treatment with 5–20 μM Quer or 1–5 mM 2-DG separately caused a concentration-dependent decrease in viable cell number, as determined by the MTT assay, and the response was augmented when the drugs were used in combination. In spite of the evident effect on proliferation, treatment with either 5–20 µM Quer or 1-5 mM 2-DG separately caused very low (less than 10 %) apoptotic effect, as measured by the frequency of cells with sub-G_1_ DNA content in flow cytometry assays. Nonetheless, apoptosis was greatly potentiated in more than additive manner when the drugs were used in combination (Fig. 1b, c). On the ground of the obtained results, the concentrations of 20 µM Quer and 5 mM 2-DG were selected for the following experiments, except when otherwise indicated. The cooperative apoptotic action between Quer and 2-DG was confirmed using the annexin V/PI assay (Fig. 1d). Moreover, the pan-caspase inhibitor z-VAD-fmk almost totally abrogated the formation of apoptotic cells (Fig. 1b–d), corroborating that cell death represents genuine caspase-dependent apoptosis. Of note, treatment with Quer plus 2-DG resulted in free PI uptake by a high proportion of cells. Nevertheless, this effect was also suppressed by z-VAD-fmk (Fig. 1e), indicating that these cells represent late apoptosis (or “secondary necrosis”) instead of a genuine necrotic response. Finally, a time-course study (3–24 h) revealed that significant more than additive drug cooperation was firstly detectable at 6 h of treatment (approx. 15 % apoptosis in the combined treatment), and increased thereafter (see Additional file 1: Fig. S1). Thus, except when otherwise indicated, 6 h was the maximum time period used for further investigation of early regulatory events.

**Fig. 2:**
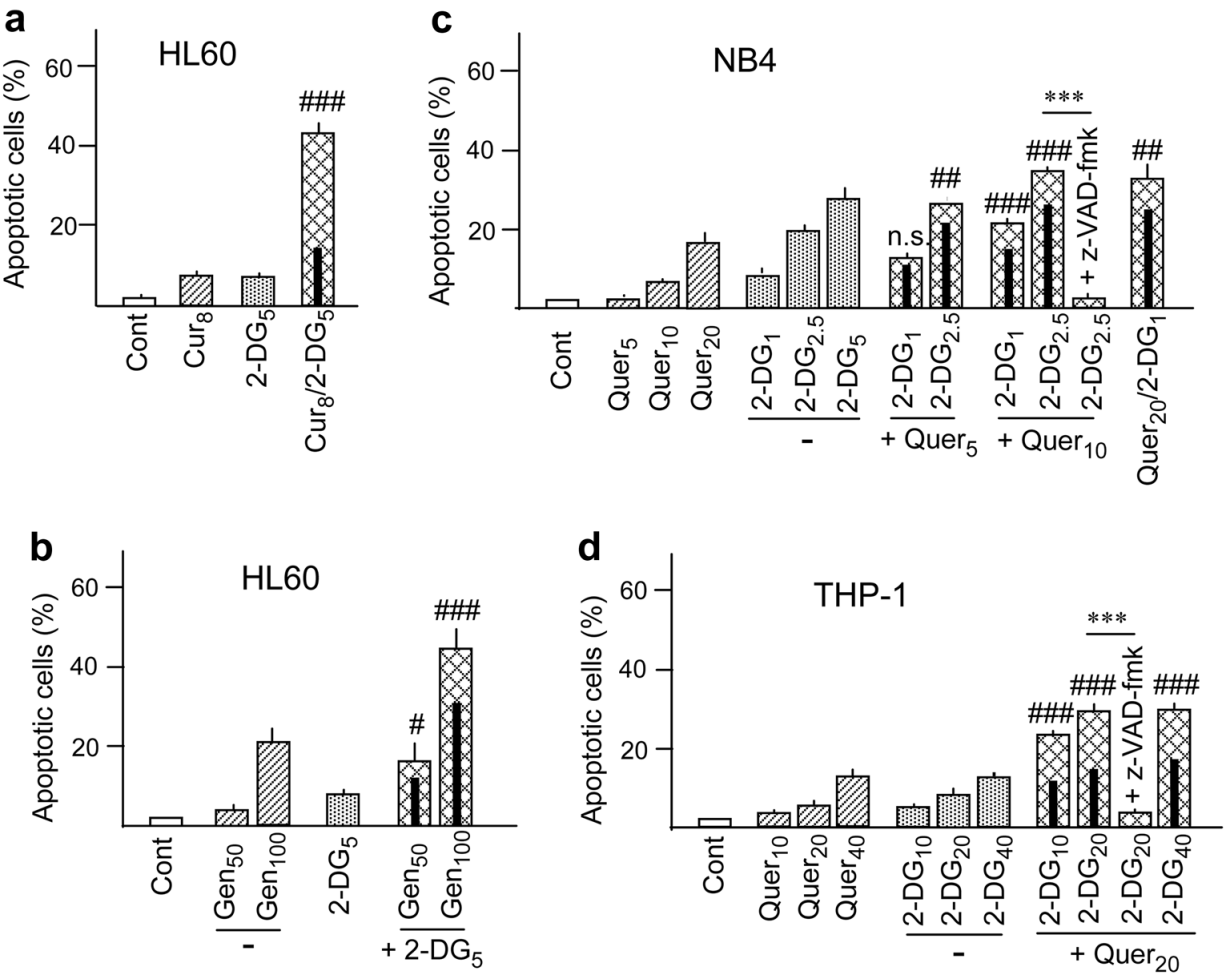
Apoptosis generation by other polyphenols, and by 2-deoxy-d-glucose and quercetin in other cell lines. (**a**, **b**) HL60 cells were incubated with the indicated concentrations of (**a**) curcumin (Cur, μM) or (**b**) genistein (Gen, μM) and 2-DG (mM), alone or in combination. **c** NB4 cells and **d** THP-1 cells were incubated with the indicated concentrations of Quer (μM) and 2-DG (mM), alone and in combination. The frequency of apoptosis (cells with sub-G_1_ DNA content) was estimated by flow cytometry. Other conditions, including pre-incubation with polyphenols in the combined treatments and symbols used in statistical analysis, were as in Fig. 1

In a new set of experiments, 2-DG was combined with Cur (8 μM) and Gen (50 μM) instead of Quer. The suitability of these concentrations for combinatory studies in leukemia cell models was established in earlier publications [21, 29]. Some of the obtained results are presented in Fig. 2a, b. Cur alone caused negligible apoptosis but cooperated with 2-DG with similar efficacy as Quer (Fig. 2a). On the other hand, the efficacy of cooperation using 50 μM Gen was very low, and the concentration had to be increased to 100 μM (which is per se moderately lethal) to obtain a more satisfactory response (Fig. 2b). The results with both Cur and Gen were corroborated using annexin V/PI analysis (see Additional file 2: Fig. S2).

In addition, we examined the possible cooperation between Quer and 2-DG in NB4 promyelocytic and THP-1 promonocytic cells. Among other biochemical aspects, these cells exhibit marked differences in the metabolic phenotype: NB4 cells are highly dependent on glycolysis, while THP-1 cells may compensate glycolysis inhibition with fatty acid β-oxidation [30]. Accordingly, Fig. 2c, d shows that NB4 cells are more susceptible and THP-1 cells less susceptible to the cytotoxic action of 2-DG than HL60 cells. The figure also indicates more than additive cooperation between Quer and 2-DG in both cell lines, although with lower efficacy than in HL60 cells. Of note, the lower apoptotic efficacy was not due to a switch to a genuine necrotic response, since free PI uptake was almost totally suppressed by the caspase inhibitor (23.3 ± 1.1 vs. 5.8 ± 0.7 % positive cells upon treatment with 10 μM Quer plus 2.5 mM 2-DG in the absence and presence of z-VAD-fmk, respectively, in NB4 cells; and 19.3 ± 1.2 vs. 4.7 ± 1.0 % upon treatment with 20 μM Quer plus 20 mM 2-DG in the absence and the presence of z-VAD-fmk, respectively, in THP-1 cells).

**Fig. 3:**
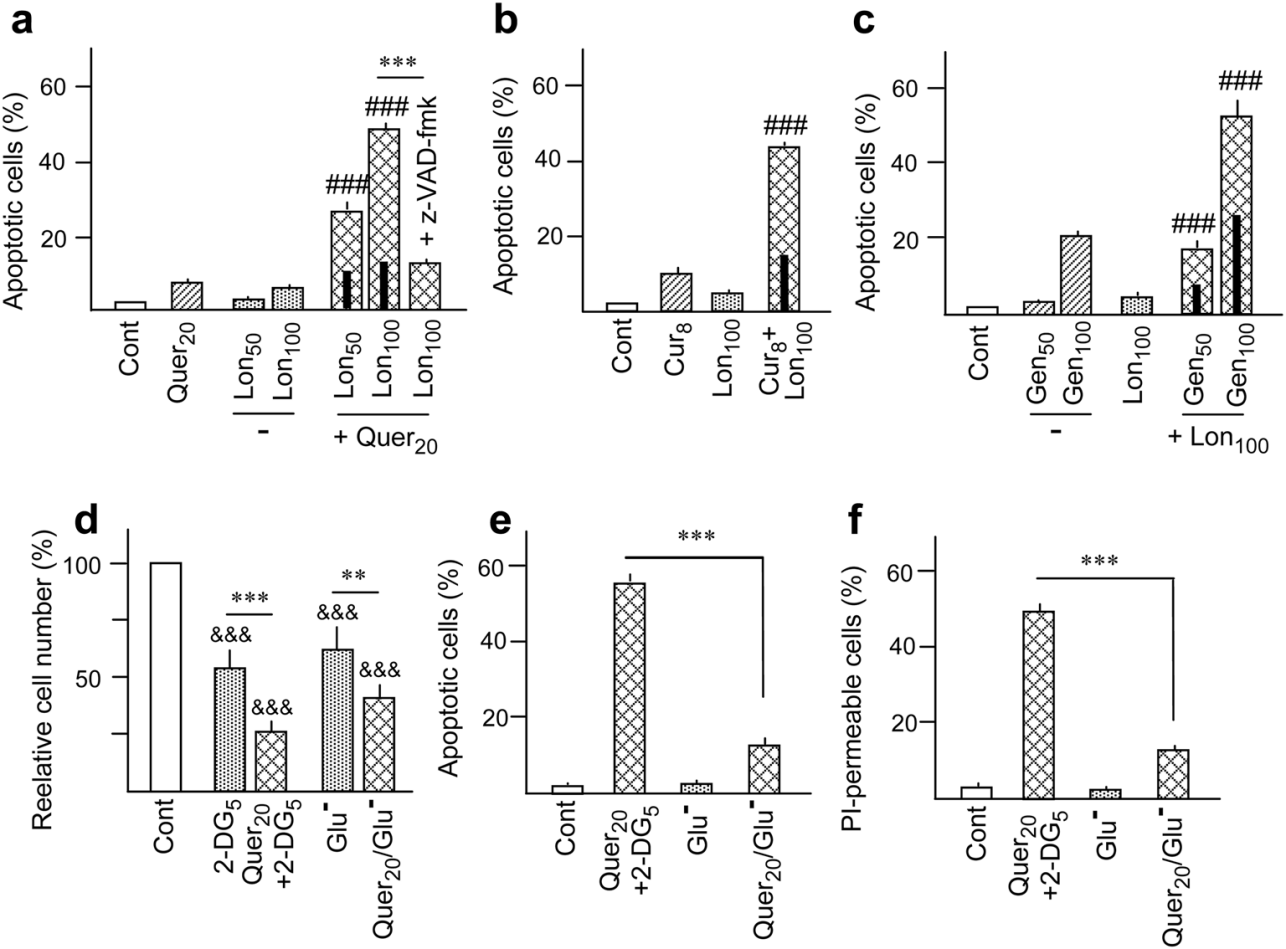
Effect lonidamine, glucose deprivation, and polyphenols on proliferation and apoptosis. (**a**–**c**) Frequency of apoptosis upon incubation of HL60 cells with the indicated concentrations (μM) of **a** Quer, **b** Cur, **c** Gen, and lonidamine (Lon), alone and in combination. (**d**–**f**) The *bar charts* indicate (**d**) the relative cell number, as an estimation of proliferation rate, (**e**) apoptosis, and (**f**) free PI uptake upon 24 h culture of HL60 cells in medium lacking glucose (Glu−), with or without Quer. Values in (**d**) are expressed in relation to cultures maintained in complete (glucose-containing) medium. In all cases, treatments with 2-DG alone or with Quer plus 2-DG are included as controls. Symbol (&) mean significant differences in relation to Cont. Other conditions, including pre-incubation with polyphenols in the combined treatments and other symbols used in statistical analysis, were as in Fig. 1

It has been reported that flavonoids such as Quer and Gen may inhibit glucose and 2-DG uptake in different cell models, including AML cells, probably by affecting glucose transporter (GLUT-1) [31, 32]. For this reason new experiments were carried out using Lon, a HKII inhibitor structurally unrelated to 2-DG. The concentrations of 50 and 100 μM Lon were adopted as adequate for combinatory studies, according to our previous publications [11, 21]. The lethality of Lon alone was very low, but efficaciously cooperated with Quer, Cur and Gen to induce apoptosis in HL60 cells, as demonstrated by the frequency of cells with sub-G_1_ DNA content (Fig. 3a–c), and confirmed in the case of Quer plus Lon by the annexin V/PI assay (see Additional file 2: Fig. S2). The protective action of z-VAD-fmk corroborated again that cell death represented caspase dependent apoptosis (Fig. 3a, c), and that the concomitant free PI uptake was attributable to late apoptosis instead of genuine necrosis (38 ± 1.7 % vs 7.2 ± 0.7 PI-permeable cells upon treatment with 20 μM Quer plus 100 μM Lon in the absence and the presence of z-VAD-fmk, respectively).

Finally, we performed a set of experiments in which, instead of being treated with 2-DG, HL60 cells were cultured for 24 h in glucose–lacking (Glu−) medium, either in the absence or the presence of Quer. Both 2-DG treatment in complete (Glu+) medium and cell incubation in Glu**−** medium resulted in partial depletion (approximately 40 % at 16 h) of intracellular ATP levels [12], and in cell proliferation inhibition (measured by cell counting), which in the case of Glu**−** was exacerbated by addition of Quer (Fig. 3d). However, cells cultured in Glu**−** were viable, and the generation of apoptosis by Quer/Glu**−** was very low, in comparison to Quer plus 2-DG in complete medium (Fig. 3e). Again, the low apoptotic rate was not compensated by a switch to a necrotic response, as indicated by the low frequency of cells with free PI uptake (Fig. 3f).

### Mitochondrial dysfunction

**Fig. 4:**
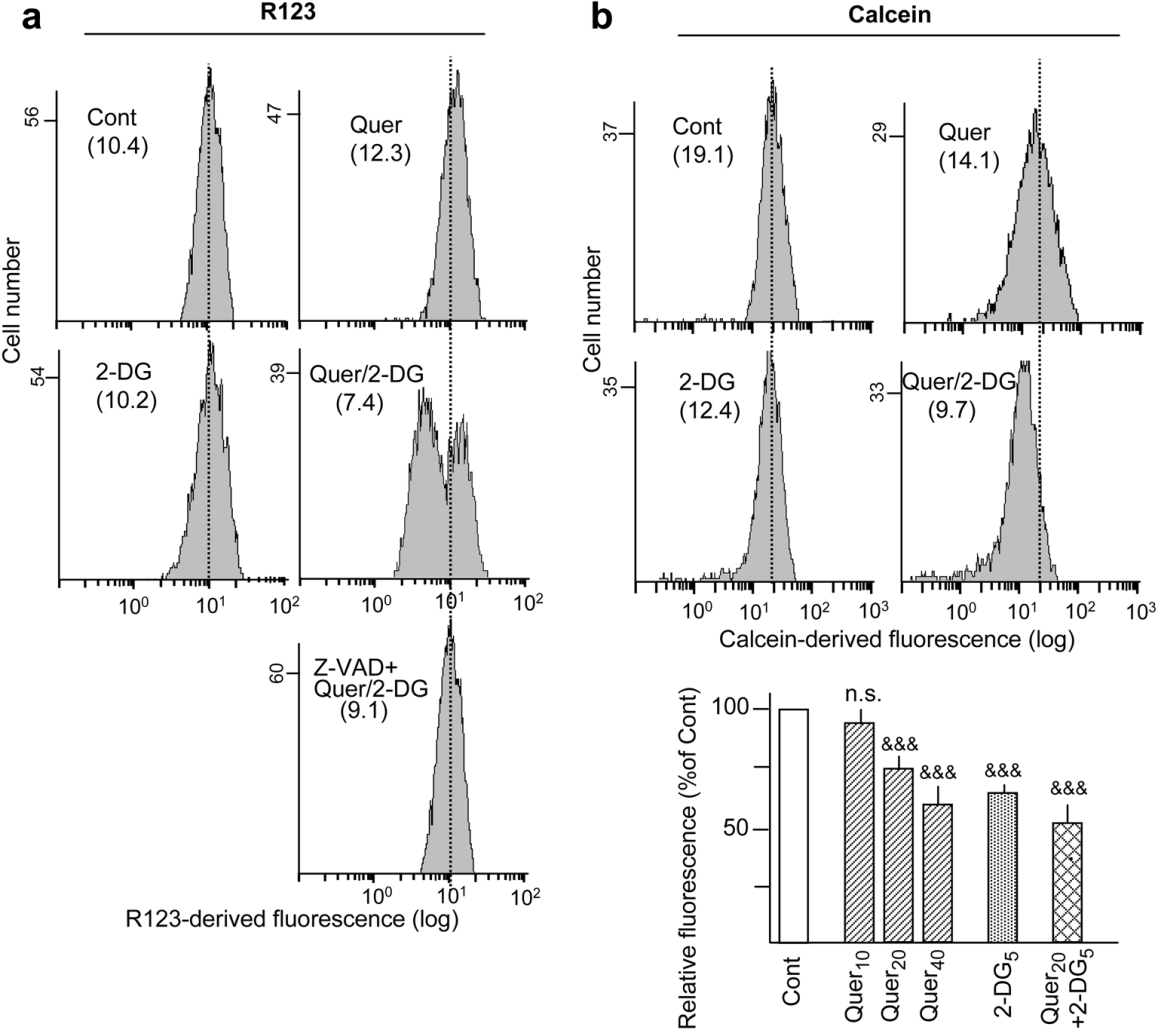
Mitochondrial transmembrane potential (ΔΨm) dissipation and inner mitochondrial membrane permeabilization (mIMP). HL60 cells were treated with Quer and 2-DG, alone or in combination. When indicated, the cells were also co-incubated with the pan-caspase inhibitor z-VAD-fmk (50 μM). **a** Flow cytometry histograms representing ΔΨm alterations at 14 h treatment with 20 μM Quer and 5 mM 2-DG, as indicated by changes in R123-derived fluorescence. Note the appearance of a discrete sub-population with low ΔΨm in the combined treatment, which disappears by application of z-VAD-fmk. **b** mIMP was measured by flow cytometry assays using the Calcein/CoCl_2_ procedure. The *bar chart* (*bottom*) indicates alterations at 4 h treatment, expressed in relation to untreated (Cont) cells. Some cytometry histograms using 20 μM Quer and 5 mM 2-DG are represented as examples (*top*). In all cytometry histograms, the numbers into parenthesis indicate the corresponding mean value, and the vertical dotted lines represent the mean value in the Cont to better discern the displacements caused by the treatments. Other conditions, including pre-incubation with Quer in the combined treatments and symbols used in statistical analysis, were as in Figs. 1 and 3

2-DG causes mitochondrial HKII inhibition and detachment and may therefore induce mIMP [7], as we previously corroborated in HL60 cells [12]. In addition, it has been reported that Quer may induce mIMP in isolated mitochondria by direct interaction with the adenine nucleotide translocase [33], although another study indicated both mIMP induction or inhibition, depending on the assay conditions [34]. In the present work, mitochondria dysfunction was firstly analyzed by measuring changes in ΔΨm after 14 h treatment with 20 μM Quer and 5 mM 2-DG, alone and in combination. The results are represented in Fig. 4a. Allowing for a slight ΔΨm increase by Quer alone, the most prominent effect was the appearance of a large subpopulation of cells with markedly low ΔΨm in the combined treatment. This subpopulation was suppressed by z-VAD-fmk, and hence likely represents the fraction of cells undergoing apoptosis. Then, a second set of experiments was carried out using the calcein/CoCl_2_ procedure, which is considered to provide a direct and more accurate determination of mIMP [35]. The results in Fig. 4b indicate that 2-DG (5 mM) and Quer (10–40 μM, in a concentration-dependent manner) caused mIMP induction, as evidenced by the decrease in calcein-derived fluorescence, and the decrease was augmented when both drugs were used in combination. Of note, this response was detected at 4 h of treatment, preceding the first manifestations of apoptosis execution (see Additional file 1: Fig. S1).

### Oxidative stress

**Fig. 5:**
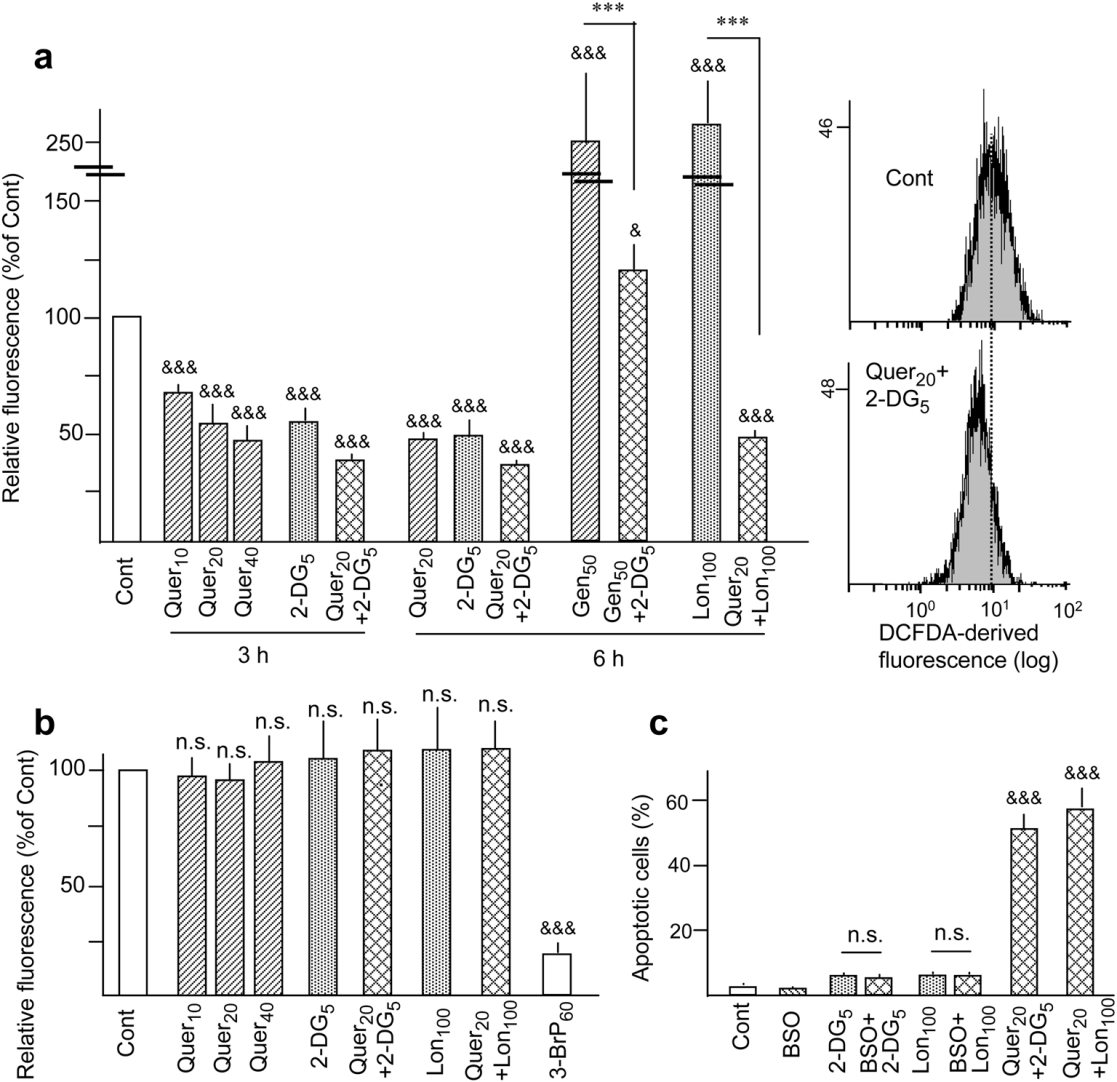
Effect of polyphenols and glycolytic inhibitors on intracellular ROS and GSH levels. **a** Intracellular accumulation of ROS in HL60 cells, as determined by flow cytometry using H_2_DCFDA, upon treatment with the indicated concentrations of Quer, Cur, Gen and Lon (μM) or 2-DG (mM), alone and in combination. The results in the bar chart are expressed in relation to untreated (Cont) cells. Cytometry histograms corresponding to untreated cells and cells incubated for 3 h with Quer plus 2-DG are presented as examples. **b** Intracellular GSH levels in HL60 cells, as determined at 6 h of treatment by monochlorobimane derivatization in luminometric assays. 3-BrP (60 μM) was used as a positive control (see Ref. [44]). The results are expressed in relation to untreated (Cont) cells (approximate GSH content, 8.5 nmol/10^6^ cells). **c** Frequency of apoptosis inHL60 cells upon incubation for 24 h with 1 mM BSO, alone and in combination with 2-DG or Lon. The combinations Quer plus 2-DG and Quer plus Lon are included as positive controls. Symbols in (**a**, **b**) indicate significant differences in relation to Cont, and in (**c**) between the indicated pairs of values (*n.s.* non significant). Other conditions, including pre-incubation with Quer in the combined treatments (**a**, **b**) and symbols used in statistical analysis, were as in Figs. 1 and 3

Although dietary polyphenols are normally considered as anti-oxidant, protective agents, there is ample evidence indicating that they may exert both anti-oxidant and pro-oxidant effects, depending on the chemical structure and treatment conditions. For instance, it was reported that Quer may either decrease [36] or increase [37, 38] ROS production in HL60 cells, and ROS increase mediated apoptosis induction [38]. For these reasons, we evaluated possible alterations in intracellular ROS accumulation after short treatments (3 h) with Quer and 2-DG, alone and in combination, using the ROS-sensitive fluorescent probe H_2_DCFDA. Some of the obtained results are presented in Fig. 5a. It was observed that 2-DG (5 mM) and Quer (10–40 μM, in a concentration-dependent manner) reduced the basal intracellular ROS content in HL60 cells, and the reduction was higher in the combined treatment. By contrast to Quer, Gen (50 μM) increased ROS levels (as previously reported [29]), but the increase was attenuated by combination with 2-DG. In a similar manner, and by contrast to 2-DG, Lon (100 μM) increased ROS content (as previously reported [11]), but this increase was totally abrogated by combination with Quer, reaching similar levels as with Quer alone. In summary, these results evidence large discrepancies in ROS production depending on the used treatment, which do not match and hence may not explain apoptosis potentiation in the combined treatments (see Figs. 1–3).

We previously reported that prolonged Quer treatment (14–24 h) reduced the intracellular GSH content in myeloid cells [20], while Gen and Cur were ineffective [21, 29]. In the present experiments we analyzed possible changes in GSH content upon short treatments of HL60 cells with Quer and the glycolytic inhibitors, using the GSH-sensitive fluorescent probe monochlorobimane. It was observed that GSH levels were not significantly affected by treatment for 3 h (data not shown) or 6 h (Fig. 5B) with 10–40 μM Quer, either alone or in combination with 2-DG (5 mM) or Lon (100 μM). Nonetheless, while these results exclude GSH as an early regulatory factor, we might not a priori exclude that a possible late depletion could have some effect on apoptosis progression. This possibility was indirectly investigated using BSO, a GSH specific synthesis inhibitor [39]. Treatment of HL60 cells with 1 mM BSO for 16 h caused a partial (35.3 ± 3.2 %) decrease in the basal GSH content, but the GSH inhibitor did not affect the proliferation rate (data not shown), nor caused cell lethality per se or in combination with 2-DG or Lon (Fig. 5c).

### Protein kinase activation

**Fig. 6:**
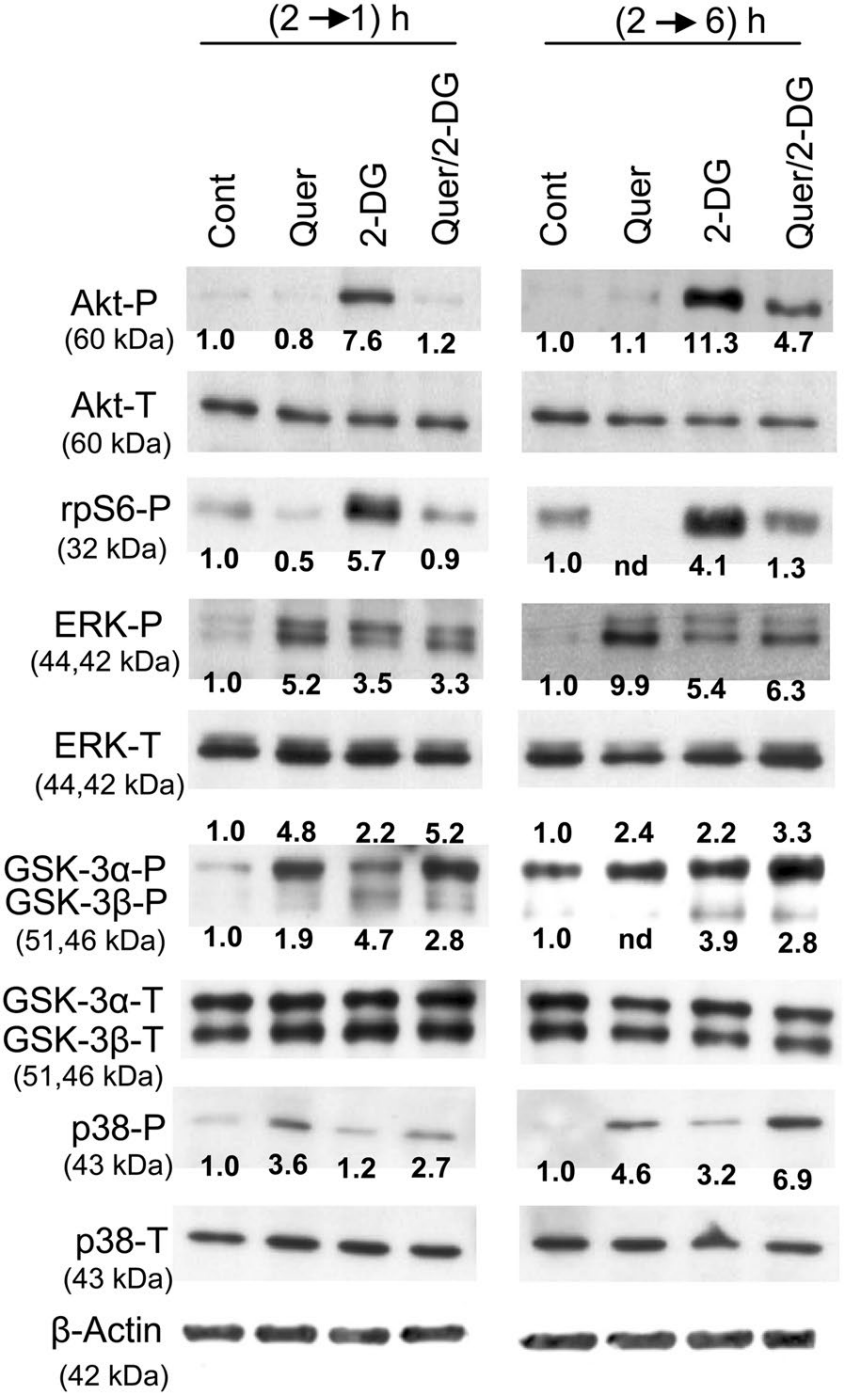
Effect of 2-deoxy-d-glucose and quercetin on protein kinase activities. The figure shows the relative levels of phosphorylated (P) and total (T) Akt, ERKs, S6 ribosomal protein (rpS6), GSK-3α,β, and p38-MAPK, and β-actin (assessed as a control of sample loading). HL60 cells were kept untreated, incubated for 1 or 6 h with 5 mM 2-DG alone, or incubated for 2 h with 20 μM Quer and then for 1 or 6 h more with or without addition of 2-DG. Whenever possible, band intensities of phosphorylated forms were measured, normalized with to the corresponding total form, and expressed in relation the Cont (arbitrary value of 1.0) (see values within the blots: *nd* not determined). ERK1/ERK2 were measured together, while GSK-3α and GSK-3β were separately analyzed. The blots are representative of one of at least three independent determinations, with qualitatively similar results. For other conditions see legend of Fig. 1

As commented above (see ‘‘Background’’ section), we hypothesized that polyphenols might potentiate the apoptotic efficacy of glycolytic inhibitors by preventing the activation of defensive kinase pathways, either PI3K/Akt and/or MEK/ERK. To examine this hypothesis, in a first set of experiments we examined Akt and ERK phosphorylation/activation upon 1 and 6 h treatment of HL60 cells with Quer and 2-DG, alone and in combination (where, as previously indicated, Quer was applied 2 h before 2-DG). We also checked the response of S6-ribosomal protein (rpS6), which is downstream Akt; of GSK3α/β, which are phosphorylated by Akt and ERK [40, 41]; and of p38-MAPK, also described as a target of quercetin or quercetin-derived analogs in leukemia cells [42, 43]. The results, presented in Fig. 6, were as follows: (i) 2-DG (5 mM) stimulated Akt and rpS6 phosphorylation/activation, and the stimulation was abrogated or greatly attenuated by Quer. (ii) By contrast, ERK phosphorylation/activation was stimulated by both Quer and 2-DG, alone and in combination. (iii) Quer and 2-DG, alone and in combination, stimulated GSK3α/β phosphorylation (Ser21/9)/inactivation. Quer alone exerted higher effect on the α isoform, while 2-DG alone stimulated both isoforms. (iv) Quer, alone or with 2-DG, stimulated p38-MAPK phosphorylation/activation, while the effect of 2-DG alone was negligible.

**Fig. 7:**
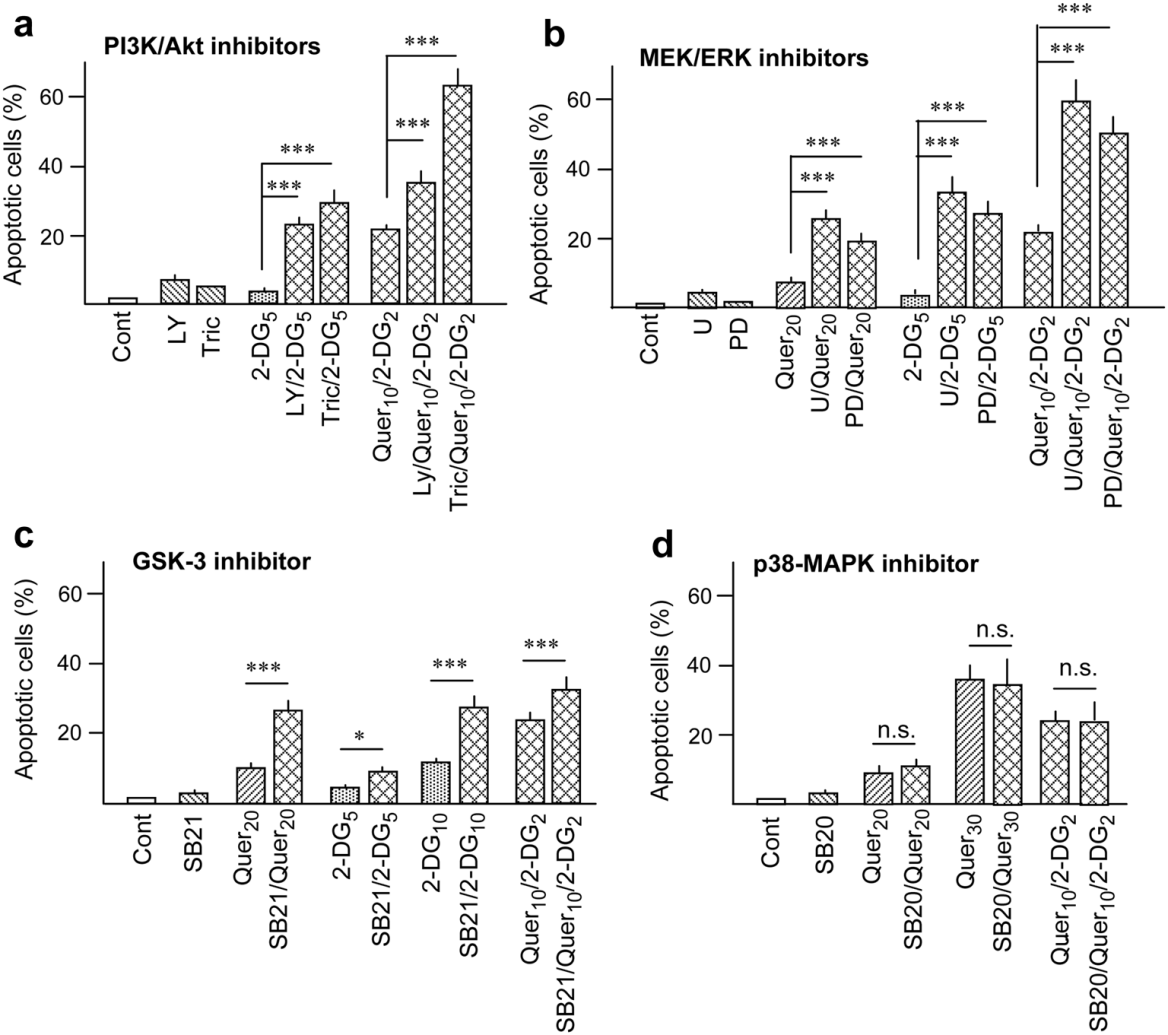
Effect of protein kinase inhibitors. The figure shows the capacity of protein kinase inhibitors to modulate apoptosis generation in HL60 cells by treatment with the indicated concentrations of Quer (μM) and 2-DG (mM), alone or the combination. As a rule, the kinase inhibitors were applied 1 h before the other drugs. **a** Effects of the PI3 K inhibitor LY294002 (LY, 30 μM) and the Akt inhibitor triciribine (Tric, 10 μM). **b** Effects of the MEK/ERK inhibitors U0126 (U, 5 μM) and PD98059 (PD, 20 μM). **c** Effects of the GSK-3 inhibitor SB216763 (SB21, 10 μM). **d** Effects of the p38-MAPK inhibitor SB203580 (SB20, 10 μM). Results are the mean ± S.D. of at least four determinations. Other conditions, including pre-incubation with Quer in the combined treatments and symbols used in statistical analysis, were as in Fig. 1

Once we examined protein kinase modulation, the potential importance of these alterations was investigated using appropriate pharmacological inhibitors (the selected concentrations being adopted from our preceding studies with AML cells [44]). The results were as follows: (i) Co-treatment with the PI3K/Akt phosphorylation/activation inhibitors LY294002 (30 μM) or triciribine (10 μM) increased the apoptotic efficacy of 2-DG alone, and also augmented the slight apoptosis obtained with the combination of low concentrations of Quer (10 μM) plus 2-DG (2 mM) (Fig. 7a). This corroborates the role of Akt as a defensive kinase, and indicates that its inhibition by Quer may be at least in part responsible for the increased apoptosis in the combined (Quer/2-DG) treatment. (ii) Co-treatment with the MEK/ERK inhibitors PD98059 (20 μM) or U0126 (5 μM) increased the apoptotic efficacy of Quer and 2-DG alone, and also augmented apoptosis by the Quer/2-DG combination (Fig. 7b). This indicates that ERK functions as a defensive kinase serving to restrain lethality by Quer and 2-DG, but may not account for the increased apoptotic efficacy in the combined treatment. (iii) Co-treatment with the GSK-3 phosphorylation inhibitor SB216763 (10 μM) augmented the lethality of Quer and 2-DG alone and (although with lower efficacy) of the Quer plus 2-DG combination (Fig. 7c), indicating that drug-provoked GSK-3 phosphorylation/inactivation plays a defensive role. (iv) Finally, activation of p38-MAPK by Quer alone or Quer plus 2-DG seemed irrelevant for apoptosis, since the lethality was not modified by the pharmacologic inhibitors SB203580 (10 μM) (Fig. 7d) or BIRB 796 (0.1 μM: data not shown).

**Fig. 8:**
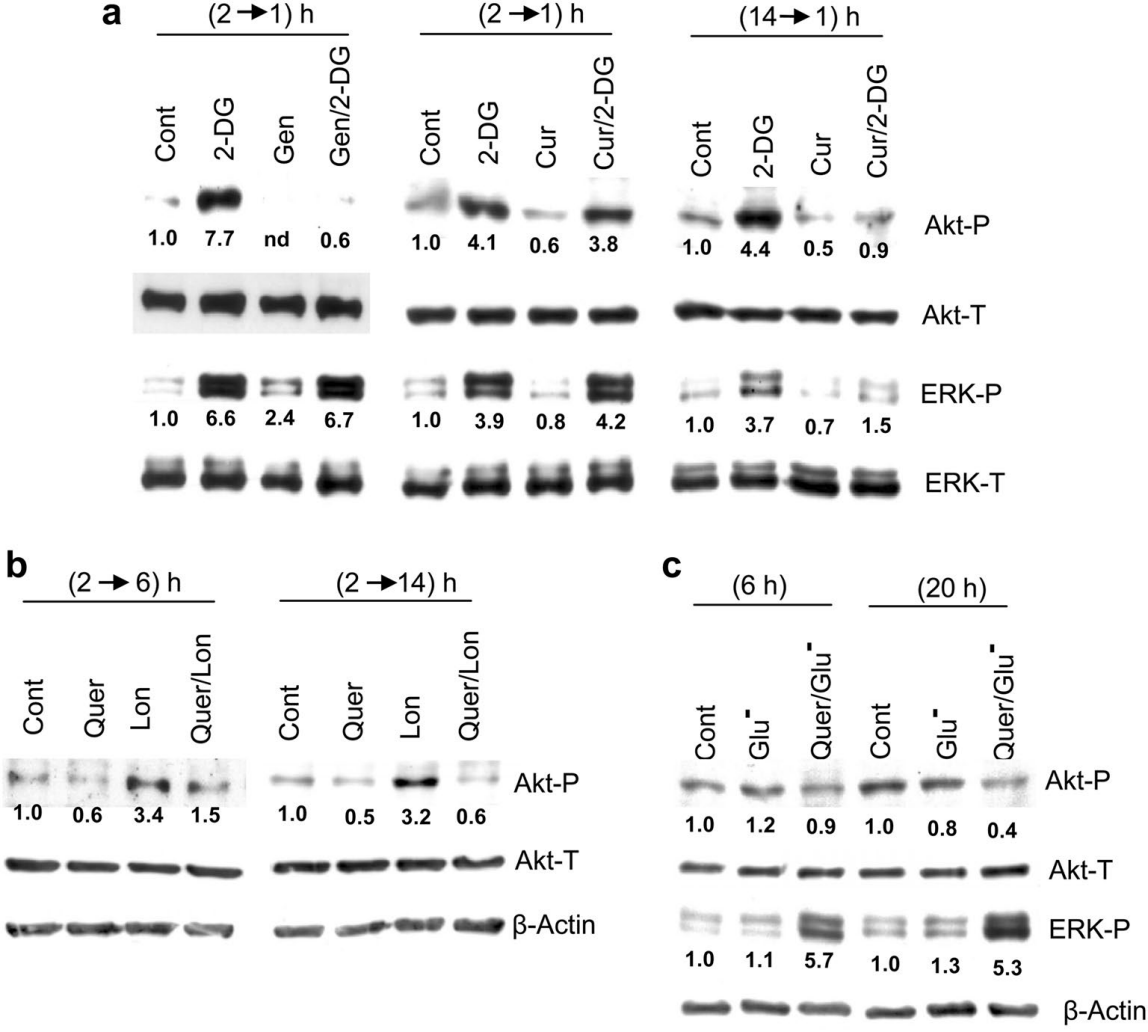
Effects of other polyphenols, lonidamine and glucose starvation on protein kinase activities. In (**a**) HL60 cells were incubated for 1 h with 5 mM 2-DG alone, or incubated for 2 or 14 h with 50 μM Gen or 8 μM Cur and then for 1 h more with or without addition of 2-DG. In (**b**) HL60 cells were incubated for 6 or 14 h with 100 μM Lon, or incubated for 2 h with Quer and then for 6 or 14 h more with or without addition of Lon. The temporal sequence is indicated as (x–y h) at the *top* of the panels. In (**c**) HL60 cells were maintained in complete (glucose-containing) medium (Cont), or incubated for the indicated time periods in glucose-lacking medium (Glu−), with or without Quer. For other conditions see legends of Figs. 1 and 6

In final set of experiments, we analyzed Akt and ERK phosphorylation using other phenolic agents and glycolytic inhibitors, as previously assayed for apoptosis (see Figs. 2 and 3). The results are represented in Fig. 8a–c, and may be summarized as follows: (i) Gen (50 μM) produced qualitatively similar effects as Quer, namely rapid abrogation of 2-DG-provoked Akt phosphorylation, and (albeit with lower intensity) stimulation ERK activation when used alone. By contrast Cur (8 μM) was ineffective at 2 h, and required longer pre-treatment to suppress Akt activation. Moreover, under these conditions Cur, which did not per se affect ERK phosphorylation, prevented the activation of this kinase by 2-DG (Fig. 8a). (ii) Lon was assayed at 6 and 14 h, since we previously showed that kinase activations by this agent are delayed in relation to 2-DG [11]. It could be observed that Quer abrogated or greatly reduced Lon-provoked Akt activation at both time periods (Fig. 8b). (iii) Finally, by contrast to the strong stimulatory effect of 2-DG, the basal Akt phosphorylation was not affected by glucose starvation (Glu−), and co-incubation with Quer either did not affect (6 h) or caused a slight late decrease (20 h) (Fig. 8c). This correlates with apparent lack of lethality of glucose-starvation, and the low apoptotic efficacy of Quer under these conditions (Glu−/Quer) in comparison to 2-DG/Quer (see Fig. 3f). By contrast, ERK phosphorylation was strongly stimulated by the combined (Glu−/Quer) treatment (Fig. 8c).

## Discussion

The present results indicate that that pre-treatment with low lethal concentrations of the flavonoid Quer strongly potentiates the anti-proliferative and apoptotic action of the glycolytic inhibitor 2-DG in HL60 AML cells. Apoptosis was assessed using different markers, namely DNA loss, phosphatidyl serine translocation, and ΔΨm decrease, and the protective action of z-VAD-fmk proves that it is in fact a caspase-mediated response. Our precedent studies demonstrated that Quer and 2-DG, as well as the other agents used here (Lon, Cur and Gen) activated apoptosis throughout the mitochondrial (“intrinsic”) executioner pathway [11, 12, 20, 21, 29]. In this study we show that 2-DG and Quer, both of them characterized as mitochondria-targeting drugs [7, 33], cause the rapid induction of mIMP (4 h), which may therefore represent a trigger or at least a necessary condition for apoptosis. Unfortunately the cause-effect relationship between mIMP and apoptosis could not be corroborated, due to the elevated toxicity of commonly used permeability transition pore modulators (cyclosporine A, bongkrekic acid) in leukemia cell models ([45], and our data not shown).

Additional experiments corroborated the cooperative effect using other polyphenols (Cur, Gen), anti-glycolytic agents (Lon), and leukemia cell models (NB4 promyelocytic, THP-1 promonocytic), although with different efficacy. For instance, Gen was less efficacious than Quer and Cur, and a moderately lethal concentration of the isoflavone was required to obtain good cooperation with 2-DG. As possible explanations, Gen causes cell arrest at G_2_/M (see Additional file 3: Fig. S3) and also stimulates myeloid cell differentiation [22], which might temporarily restrain the trigger of the apoptotic response. In the same manner, the efficacy of cooperation between Quer and 2-DG was lower in NB4 and THP1 than in HL60 cells. This might be explained by intrinsic differences in molecular and biochemical properties and in the maturation stage of these cell lines. For instance, in addition to the above indicated different susceptibility to 2-DG, THP-1 promonocytic cells are more resistant to Quer [46] (an in our experience also to other cytotoxic agents) than the less mature HL60 and NB4 cells. On the other hand, the finding that Quer plus 2-DG and Quer plus Lon induced apoptosis with similar efficacy allows to exclude that apoptosis potentiation may be a trivial consequence of polyphenol-provoked inhibition of GLUT 1 activity and 2-DG uptake, and hence of subsequent biochemical responses (e.g., 2-DG-provoked activation of defensive kinases, as discussed later). By contrast, the apoptotic efficacy of Quer was only marginally increased when 2-DG treatment was substituted by glucose starvation. This indicates that the partial ATP depletion provoked by both treatments may affect cell proliferation, but it is not a determinant of drug lethality. Finally, a cautionary note must be expressed. We centered the attention on the apoptotic response, and for convenience (better observation of drug cooperation) selected sub-lethal drug concentrations. Nonetheless these concentrations caused appreciable anti-proliferative effects, as measured by the MTT assay (Fig. 1a) or by cell counting (the proliferation inhibition rates at 24 h, in relation to the control, were: 43 % for 5 mM 2-DG; 5 % by 100 μM Lon; 44 % by 20 μM Quer; 21 % by 8 μM Cur; and 65 % by 50 μM Gen). This may be explained in some cases by cell cycle disruption (e.g., almost total G_2_/M arrest by Gen, and with lower intensity by Quer: see Additional file 3: Fig. S3). The molecular mechanisms responsible for Gen-provoked cycle arrest were investigated in a preceding article [22]. In addition, a possible activation of autophagy, which opposes apoptosis, may not be discarded.

The generation of apoptosis and/or necrosis by anti-tumour drugs is frequently associated to oxidative stress, two manifestations of which are the increase in intracellular ROS accumulation and the decrease in anti-oxidant molecules such as GSH. For instance, we earlier demonstrated that the potentiation of arsenic trioxide (Trisenox)-provoked apoptosis by Lon and Gen was mediated by the stimulation of ROS production [11, 29], probably due to the interference of Lon and Gen with the mitochondrial respiratory chain [47, 48]. On the other hand, 2-DG was reported to decrease ROS [49, 50], while Quer may exert either inhibitory [33] stimulatory [37, 38] effects. Our present results confirm the drug-dependent variability, and above all prove that the increase in lethality in the combined treatments (Quer plus 2-DG and Quer plus Lon) may not be adequately explained by ROS over-production. Thus, Quer not only reduced the basal ROS content, but exacerbated the decrease caused by 2-DG, and reversed the increase caused by Lon to levels lower than in untreated cells. In another study we observed that prolonged (14–24 h) treatment with Quer decreased the intracellular GSH content, and as a consequence potentiated the lethality of the GSH-sensitive drug arsenic trioxide [20]. However a regulatory role of GSH may be excluded in the present conditions. In fact, Quer did not cause early changes in GSH content, and the impact on apoptosis of a potential long-term alteration may be discarded, in view of the lack of effects of GSH specific inhibitor BSO.

Finally, the present results corroborate the capacity of 2-DG and Lon to stimulate Akt activation in AML cells, and demonstrate that the stimulation is abrogated or attenuated by co-treatment with Quer, Gen and Cur, which at the same time potentiate apoptosis. The cause-effect relationship between Akt inhibition and apoptosis potentiation was supported by experiments using standard pharmacologic protein kinase inhibitors, and was also strengthened by the results obtained using glucose-free medium, where the lack of Akt activation correlates with poor Quer lethality. There are nevertheless some quantitative differences: thus, Akt activation by Lon was delayed in relation to 2-DG; a prolonged pre-treatment with Cur (14 h) was required to abrogate 2-DG-provoked Akt activation; and 50 μM Gen sufficed to block 2-DG-provoked Akt activation, although the pro-apoptotic action of this Gen concentration was poor. The discrepancies might be explained by the particular action mechanisms of the used drugs, and also indicate that Akt inhibition is an important factor but not the only one accounting for apoptotic potentiation in the combined treatments. Noteworthy, prolonged pre-treatment with Cur also abrogated 2-DG-provoked ERK activation, indicating that at least in some circumstances this kinase also regulates apoptosis potentiation in the combined treatments. Nonetheless the function of ERKs in the present experiments is less clear, mainly because the disparity of effects caused by polyphenols. Thus, ERK phosphorylation was strongly stimulated by Quer, slightly stimulated by Gen, and unaffected by Cur. Of note, co-treatment with MEK/ERK inhibitors increased the apoptotic efficacy of Quer alone. It seems therefore that ERK activation serves to restrain excessive polyphenol toxicity, in the same manner as 2-DG toxicity. In addition Quer, alone or with 2-DG, caused p38-MAP activation, but this response seems irrelevant for apoptosis as judged by the null effect of kinase inhibitors. This conclusion is consistent with results obtained by other authors using a Quer analogue [43]. Finally, earlier reports indicated that 2-DG elicits Akt-dependent GSK-3β phosphorylation [13] while quercetin increases [51] or does not affect [52] kinase phosphorylation. Using pharmacologic inhibitors, other articles proved that GSK-3 regulates cell growth and/or apoptosis in leukemia cells [53–55]. Our present results indicate that Quer and 2-DG cause hyper-phosphorylation (Ser21/9)/inactivation of GSK-3α/β in HL60 cells, although with certain drug-specificity. Thus, Quer (which only activated ERKs) preferentially stimulated the α isoform, while 2-DG (which activated Akt and ERKs) stimulated both α and β isoforms. Most studies in the literature only centered the attention on GSK-3β, but some reports call attention on the functional importance of the α isoform. As an example, GSK-3α knock-down reduced proliferation and caused spontaneous apoptosis [56], and potentiated bortezomib-induced toxicity [57], in leukemia cells. Our results show that the GSK-3 inhibitor SB216763 (10 μM) does no cause per se significant lethality, but potentiates apoptosis induction by Quer and 2-DG, alone and in combination, in HL60 cells. Since as commented above Quer preferentially stimulates GSK-3α, we might postulate that this isoform is the most important for regulation of Quer lethality. Unfortunately the lack of specificity of the up to date available pharmacologic inhibitors, and the difficulty to perform satisfactory knock-down procedures in the used leukemia cell model, impeded us until now to obtain more clear conclusions.

## Conclusions

De-regulation of the MEK/ERK and especially the PI3K/Akt pathway are among the most frequent alterations associated to the generation of the tumour phenotype, as well as to the intrinsic or acquired resistance of tumour cells to anti-cancer treatments. Dietary phenolic agents are in general well tolerated, and might therefore be preferable to approach the problem instead of synthetic pharmacologic drugs. The present results indicate that co-treatment with low concentrations of selected polyphenols increase the apoptotic efficacy of the glycolytic inhibitors 2-DG and Lon in human acute AML cell models, and that this effect may be at least in part explained by the prevention of defensive protein kinase activation, mainly Akt and in some circumstances ERKs. Of course, we did not attempt performing an exhaustive study: in fact, polyphenols affect multiple biochemical and molecular mechanisms other than Akt inhibition [16–18], 2-DG is a dual glycolysis and N-glycosylation inhibitor [4], and Lon inhibits glycolysis but also lactate transport (causing intracellular acidification) and mitochondrial respiration [5, 48]. In spite of the conceptual and technical limitations we believe that our in vitro study may offer some ideas to improve the efficacy of this potentially important group of anti-tumour drugs.

## Additional files

**Additional file 1: Fig. S1.** Time-course generation of apoptosis by Quer and 2-DG. HL60 cells were treated for the indicated time periods with 20 μM Quer and 5 mM 2-DG, alone and in combination. Apoptosis is given by the frequency of cells with sub-G_1_ DNA content. For other conditions, including pre-incubation with Quer in the combined treatments, see legend of Fig. 1 in the main text.

**Additional file 2: Fig. S2.** Apoptosis generation by several polyphenols and glycolytic inhibitors, as determined by the annexin V/PI assay. The histograms show the frequency of early (Annexin V^+^/PI^**−**^) or late (Annexin V^+^/PI^+^) apoptotic cells, upon 24 h treatment of HL60 cell cultures with 100 μM Gen and 8 μM Cur, alone and in combination with 5 mM 2-DG, or with 100 μM Lon, alone or in combination with 20 μM Quer. Other conditions, including the pre-incubation with polyphenols in the combined treatments, were as in Fig. 1. in the main text.

**Additional file 3: Fig. S3. **Cell cycle phase distribution. Representative flow cytometry histograms and frequency of cells at the different cycle phases in exponentially-growing untreated HL60 cell cultures (Cont), in cultures treated for 24 h with 5 mM 2-DG, 100 μM Lon, 20 μM Quer, 8 μM Cur, and 50 μM Gen, and in cultures incubated for 24 h in the absence of glucose (Glu-). For simplicity, the subpopulations of cells with sub-G_1_ DNA content (apoptotic) are not represented.

## Abbreviations

Akt: protein kinase B
AML: acute myeloid leukemia
BIRB 796: 1-5-*tert*-butyl-2-*p*-tolyl-2*H*-pyrazol-3-yl)-3-[4-(2-morpholin-4-yl-ethoxy)naphthalen-1-yl] urea
BSO: d l-buthionine-*S,R*-sulfoximine
Calcein-AM: calcein *O,O*’-diacetate tetrakis(acetoxymethyl) ester
Cur: curcumin
2-DG: 2-deoxy-d-glucose
ERK: extracellular signal-regulated kinase
FITC: fluorescein isothiocyanate
Gen: genistein
GSH: reduced glutathione
H_2_DCFDA: dichlorodihydrofluorescein diacetate
Lon: lonidamine
LY294002: 2-(4-Morpholinyl)-8-phenyl-4H-1-benzopyran-4-one
MAPK: mitogen-activated protein kinase
MEK: mitogen-induced extracellular kinase/extracellular signal-regulated kinase
mIMP: mitochondrial inner membrane permeability
MTT: 3(4,5-dimethyl-2-thiazolyl)-2,5-diphenyl-2*H*-tetrazolium bromide
PBS: phosphate buffered saline
PD98059: 2′-amino-3′-methoxyflavone
PI3K: phosphatidylinositol 3-kinase
PI: propidium iodide
R123: rhodamine 123
ROS: reactive oxygen species
SB203580: 4-(4-fluorophenyl)-2-(4-methylsulfinylphenyl)-5-(4-pyridyl)-1H-imidazole
SB216763: 3(2,4-dochlorophenyl)-4-(1-methyl-1H-indol-3-yl)-1H-pyrrole-2,5-diione
Triciribine: 5-dihydro-5-methyl-1-β-d-ribofuranosyl-1,4,5,6,8-pentaazaacenaphtylen-3-amine hydrate
Quer: quercetin
U0126: 1,4-diamino-2,3-dicyano-1,4-*bis*(2-aminophenylthio)butadiene
z-VAD-fmk: Z-Val-Ala-Asp(OMe)-CH_2_F
