# Supplementary figures and images for: Selected polyphenols potentiate the apoptotic efficacy of glycolytic inhibitors in human acute myeloid leukemia cell lines. Regulation by protein kinase activities
Source: Cancer Cell Int. 2016 Sep 7;16:70. doi: 10.1186/s12935-016-0345-y (PMC5015235; doi:10.1186/s12935-016-0345-y)

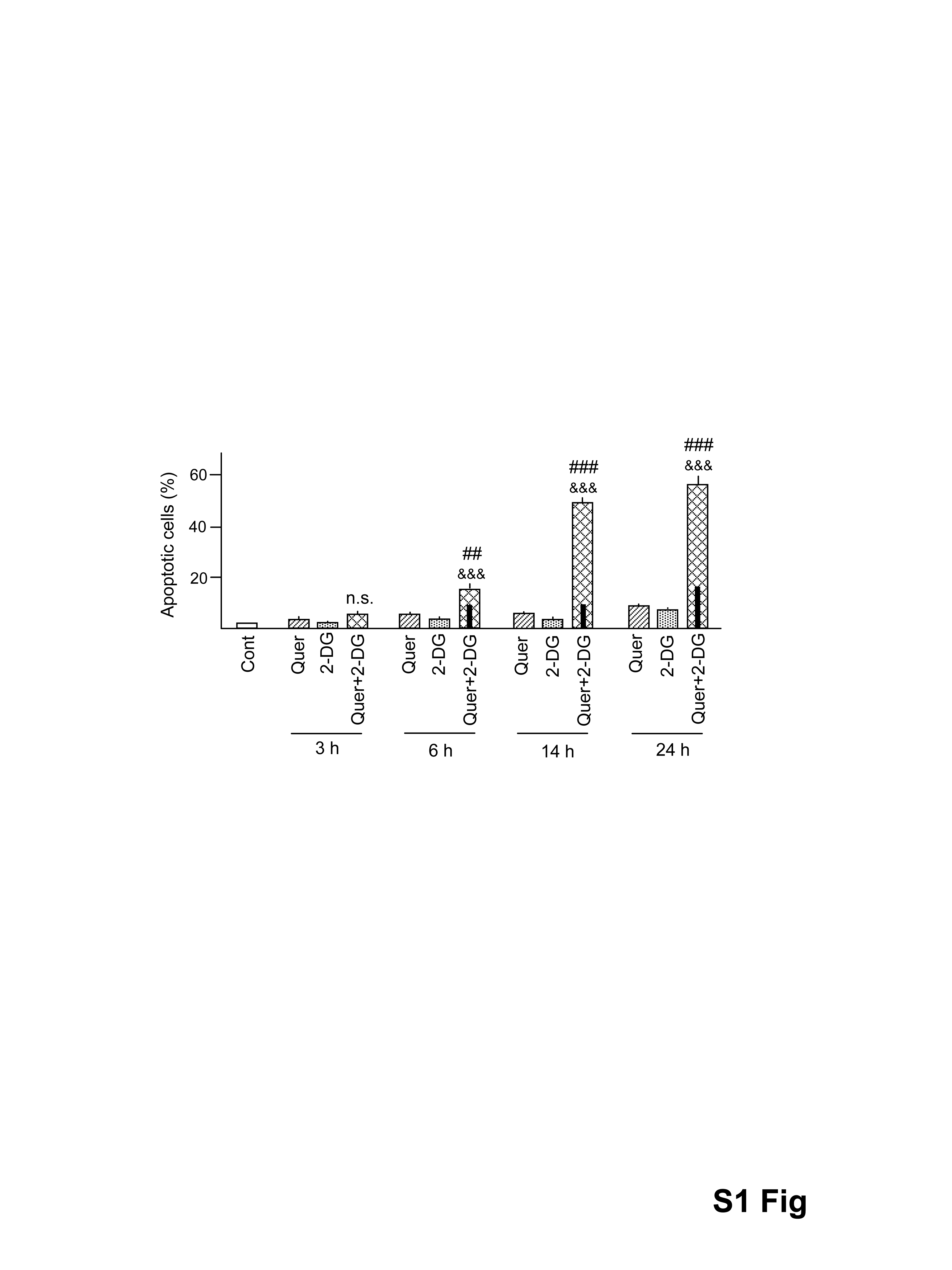

Supplement: Supplementary file 1 — Additional file 1: Fig. S1. Time-course generation of apoptosis by Quer and 2-DG. HL60 cells were treated for the indicated time periods with 20 μM Quer and 5 mM 2-DG, alone and in combination. Apoptosis is given by the frequency of cells with sub-G1 DNA content. For other conditions, including pre-incubation with Quer in the combined treatments, see legend of Fig. 1 in the main text. [file 12935_2016_345_MOESM1_ESM.tif]

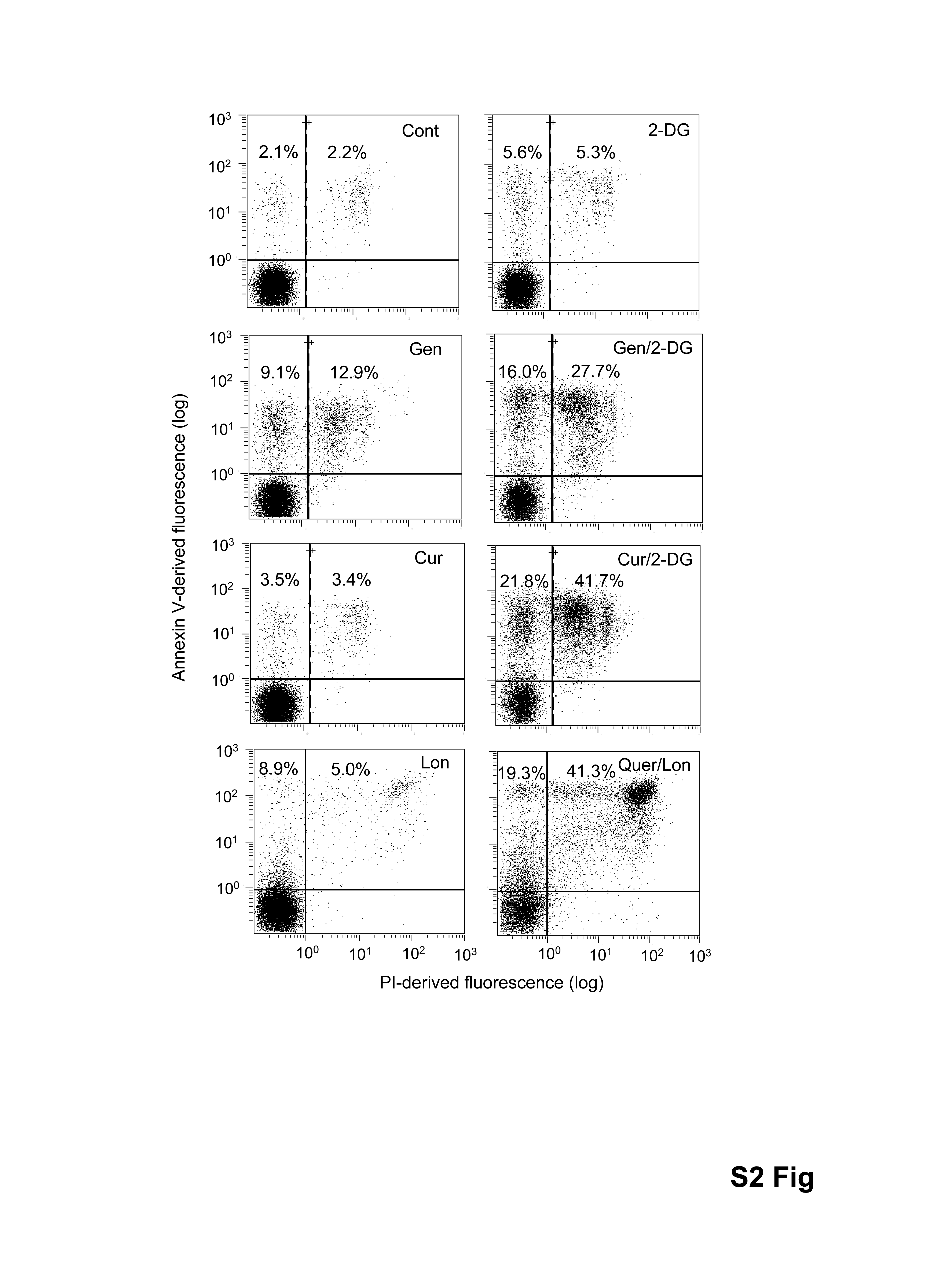

Supplement: Supplementary file 2 — Additional file 2: Fig. S2. Apoptosis generation by several polyphenols and glycolytic inhibitors, as determined by the annexin V/PI assay. The histograms show the frequency of early (Annexin V+/PI−) or late (Annexin V+/PI+) apoptotic cells, upon 24 h treatment of HL60 cell cultures with 100 μM Gen and 8 μM Cur, alone and in combination with 5 mM 2-DG, or with 100 μM Lon, alone or in combination with 20 μM Quer. Other conditions, including the pre-incubation with polyphenols in the combined treatments, were as in Fig. 1. in the main text. [file 12935_2016_345_MOESM2_ESM.tif]

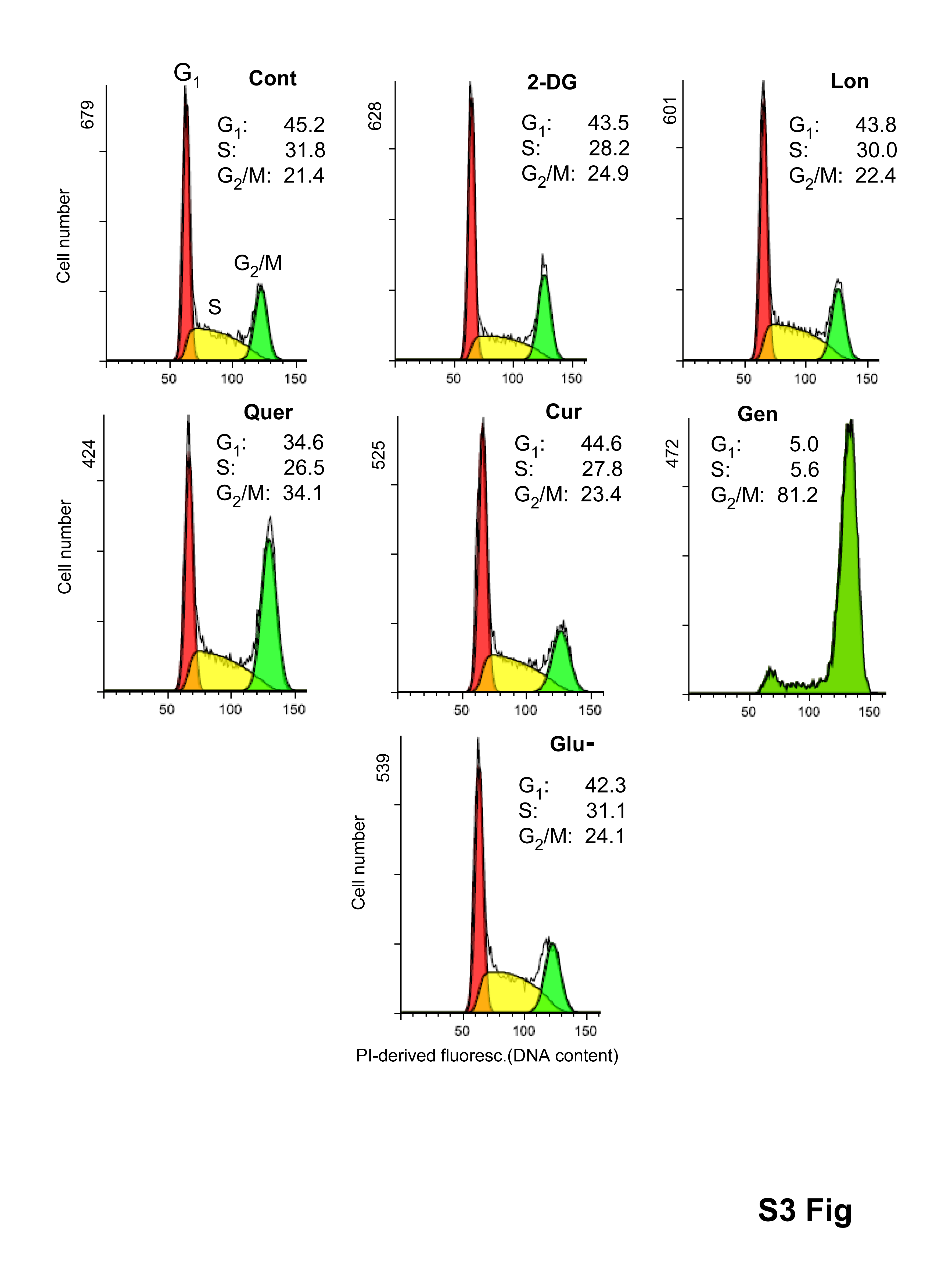

Supplement: Supplementary file 3 — Additional file 3: Fig. S3. Cell cycle phase distribution. Representative flow cytometry histograms and frequency of cells at the different cycle phases in exponentially-growing untreated HL60 cell cultures (Cont), in cultures treated for 24 h with 5 mM 2-DG, 100 μM Lon, 20 μM Quer, 8 μM Cur, and 50 μM Gen, and in cultures incubated for 24 h in the absence of glucose (Glu-). For simplicity, the subpopulations of cells with sub-G1 DNA content (apoptotic) are not represented. [file 12935_2016_345_MOESM3_ESM.tif]
